# Identification and *P1* genetic evolutionary analysis of duck hepatitis a virus type 3 in selected areas of Jiangsu, China in 2024

**DOI:** 10.1016/j.psj.2026.106824

**Published:** 2026-03-19

**Authors:** Jiawen Yu, Tingting Zhang, Zitong Guo, Yunwang Liu, Mengxue Yuan, Zijun Yang, Xibo Wang, Xingdong Song, Shijin Jiang, Liangmeng Wei

**Affiliations:** a Sino-German Cooperative Research Centre for Zoonosis of Animal Origin of Shandong Province, Shandong Provincial Key Laboratory of Zoonoses, College of Veterinary Medicine, Shandong Agricultural University, Panhe Street 7, Tai’an 271000, China; b Collaborative Innovation Center for the Origin and Control of Emerging Infectious Diseases, School of Clinical and Basic Medical Sciences, Shandong First Medical University & Shandong Academy of Medical Sciences, Jinan 250024, China; c College of Life Sciences, Shandong Agricultural University, Tai’an, China; d Shuyang Zhongke Poultry Breeding Co., Ltd., Suqian 223800, China

**Keywords:** Duck hepatitis A virus type 3, *P1*/*VP0*/*VP3*/*VP1* gene, Genetic evolutionary analysis

## Abstract

Duck hepatitis A virus type 3 (DHAV-3) is a widespread, highly lethal pathogen in East Asian ducklings. To investigate its molecular characteristics in Jiangsu, China, we performed PCR on 103 liver samples from deceased ducklings and isolated 14 DHAV-3 strains. Phylogenetic analysis of *P1, VP0, VP3*, and *VP1*, showing that the isolates shared more than 95.1% of nucleotide identities and more than 98.3% of amino acid identities, indicated close phylogenetic relationships among the isolates. Compared with reference strains, the isolates shared a high identity with strains from Shandong, Anhui, Chongqing, and Henan Provinces, but a lower identity with strains from South Korea and Vietnam, which suggests distinct geographical clustering. Phylogenetic analysis also confirmed that all isolates clustered into GI and were distinct from South Korean and Vietnamese strains in GII, thereby indicating significant genetic divergence. Meanwhile, genetic variation analysis of *VP1* and protein structure prediction for the isolates revealed amino acid mutations at positions 141 and 190 in some isolates, and at positions 141 and 207 in certain reference strains, which correlated with phylogenetic relationships. The three-dimensional structure of the VP1 protein was conserved overall, with localized alterations in mutated regions. Altogether, DHAV-3 strains in Jiangsu showed high genetic conservation but distinct geographical clustering, and the identified *VP1* variations warrant further investigation. This article elucidated DHAV-3′s molecular characteristics in Jiangsu and provided critical data for tracking viral evolution and updating regional vaccine strategies.

## Introduction

Duck viral hepatitis (**DVH**) is an acute, highly lethal disease in ducklings. Its clinical manifestations include neurological signs such as opisthotonos, and necropsy shows hemorrhagic foci of varying sizes in the liver. DVH is classified into three types: type I, type II, and type III. Type I is caused by duck hepatitis A virus (**DHAV**), whereas type II and III are caused by duck astrovirus 1 and duck astrovirus 2 (Yugo et al., 2016), respectively. Those three viruses exhibit no cross-antigenic relationships (Toth 1969; Haider and Calnek, 1979). Currently, DHAV, the primary pathogen responsible for DVH outbreaks, causes substantial economic losses to the global duck farming industry.

DHAV is the only member of the genus *Avihepatovirus* within the family Picornaviridae (Wen et al., 2018). The viral nucleocapsid is a symmetric icosahedron, and the non-enveloped virions measure approximately 20–40 nm in diameter. The DHAV genome, a single-stranded positive-sense RNA approximately 7.7 kb in length, exhibits characteristics of picornaviruses. It contains a large open reading frame (Yugo et al., 2016), a 5′ untranslated region (**UTR**), and a 3′ UTR with a poly(A) tail (Li et al., 2013; Ma et al., 2015). The viral 5′ UTR harbors regulatory elements involved in viral gene expression and an internal ribosomal entry site that initiates the translation of downstream proteins. The 3′ UTR—the longest among picornaviruses—features multiple hairpin structures. The open reading frame encodes a large polyprotein that is cleaved into both structural and non-structural proteins by viral proteases; protease 2A cleaves the polyprotein into structural polyprotein P1 and nonstructural polyproteins P2 and P3. Subsequently, protease 3C processes P1 into VP0, VP3, and VP1; P2 into 2A1, 2A2, 2A3, 2B, and 2C; and P3 into 3A, 3B, 3C, and 3D (Probst et al., 1998). The three structural proteins VP0, VP3, and VP1 mediate viral entry by specifically binding to receptors on the host cell surface. Compared with other regions of the viral genome, the genes encoding those structural proteins exhibit higher mutation frequencies, which directly determine viral antigenicity.

Based on serological and phylogenetic analyses, DHAV is classified into three serotypes and their corresponding genotypes: DHAV-1, DHAV-2, and DHAV-3 (Tseng and Tsai, 2007; Fehér et al., 2021). DHAV-1, the classical serotype, has spread widely across Eurasia and Africa since first being identified in the United States, with cases reported in the United Kingdom, Poland, China, and Egypt (Fehér et al., 2021). DHAV-2 is primarily endemic to Taiwan, China (Tseng and Tsai, 2007), with recent reports in India (Rajendran et al., 2023). Meanwhile, DHAV-3 has shown increasing prevalence in South Korea, China, and Vietnam (Kim et al., 2007; Liu et al., 2011; Doan et al., 2016). Since 2013, DHAV-3 has emerged as the predominant strain circulating in mainland China. A multi-province epidemiological survey in China demonstrated that DHAV-1 and DHAV-3 accounted for 38% and 49% of tested DVH samples, respectively (Ye et al., 2023), thereby indicating that DHAV-3 is gradually becoming the dominant epidemic strain in the country. For instance, the detection rate of DHAV-3 in Shandong Province has been reported to reach 57.1% (Zhang et al., 2017). As a key waterfowl production Province in eastern China with high duck population density, Jiangsu plays a critical role in the national poultry industry. However, systematic studies on the current prevalence and strain characteristics of DHAV-3 in Jiangsu Province remain scarce. That gap is notable given the recurrent mutations identified at key amino acid sites within its structural proteins (Li et al., 2013; Ma et al., 2015; Doan et al., 2016; Fehér et al., 2021), which may alter viral properties and compromise vaccine efficacy. Therefore, a comprehensive study of DHAV-3′s prevalence, genetic evolution, and molecular variation in Jiangsu Province is crucial for optimizing regional prevention and control strategies.

Our study aimed to investigate the prevalence of DHAV-3 in Jiangsu in 2024, clarify the genetic evolutionary characteristics of its *P1, VP0, VP3*, and *VP1* and the mutation profile of the VP1 protein, and provide experimental data for optimizing regional DHAV-3 vaccination strategies and tracking viral cross-regional transmission.

## Materials and methods

### Sample collection and preservation

In 2024, a total of 103 liver samples were collected from diseased ducks on commercial farms in major duck-producing regions of Jiangsu, including Lianyungang, Huai’an, Suqian, and Yancheng. The affected ducklings exhibited typical clinical signs of DVH, such as opisthotonos and liver petechial hemorrhages. Samples confirmed to be DHAV-3-positive by reverse transcription polymerase chain reaction (**RT-PCR**) were cryopreserved at −80°C for subsequent molecular analyses.

### Primer design and synthesis

The primers used were designed based on published DHAV-3 sequences (Zhang et al., 2017). Primers DHAV-3-F and DHAV-3-R were used to detect DHAV-3, and A-DHAV-3-F, A-DHAV-3-R, B-DHAV-3-F, and B-DHAV-3-R were used to amplify the full-length *P1* gene sequence. All primers were synthesized by Tsingke Biotechnology Co., Ltd. (Shanghai, China). Information regarding primers is detailed in Table 1.

**Table 1:** Primer sequences used.

Primer name	Amplification product (bp)	Primer sequence (5′–3′)
DHAV-3-F	720	TGTGCCTGAGAAGCGGGATA
DHAV-3-R		CCGAAAGTGGAGATTAGGTG
A-DHAV-3-F	1,224	GACCTTTGGAAGCCAGTTTA
A-DHAV-3-R		CATCACAGGCACGAACAAGT
B-DHAV-3-F	1,516	CACACTGCCTGATAGGGTCG
B-DHAV-3-R		CTGGCTTCCAAAGGTCAATAA

### RNA extraction and RT-PCR identification

Liver tissue samples from deceased ducks were homogenized (60 Hz, 6 min) with 500 μL 0.9% physiological saline and 3–5 autoclaved grinding beads. The homogenate was centrifuged at 13,687 × *g* for 2 min, and the supernatant was collected for RNA extraction. Total RNA was extracted using the FastPure Cell/Tissue Total RNA Isolation Kit V2 (Vazyme, Nanjing, China) according to the manufacturer’s instructions. cDNA was subsequently synthesized using the HiScript II Q RT SuperMix (Vazyme, Nanjing, China). PCR amplification was performed using the synthesized cDNA as the template. Each 50 μL of reaction mixture contained 25 μL of 2 × Rapid *Taq* Master Mix (Vazyme, Nanjing, China), 2.5 μL of each of DHAV-3-F and DHAV-3-R primers (10 μM), 2 μL of cDNA, and 18 μL of nuclease-free water. In PCR, initial denaturation was performed at 95°C for 5 min; next, 35 cycles of denaturation at 95°C for 15 s, annealing at 55°C for 35 s, and extension at 72°C for 35 s; and a final extension was performed at 72°C for 5 min. The PCR products were analyzed by electrophoresis on a 1% agarose gel and visualized using a gel imaging system.

### *P1* gene amplification and sequencing

*P1* gene sequences were amplified from DHAV-3-positive samples with primer pairs A-DHAV-3-F/A-DHAV-3-R and B-DHAV-3-F/B-DHAV-3-R. Reaction system and thermal cycling conditions were identical to those described in the “RNA Extraction and RT-PCR Identification” section. PCR products were analyzed using 1% agarose gel electrophoresis and visualized via a gel imaging system. Target bands were excised, and the DNA was purified using the FastPure Gel DNA Extraction Mini Kit (Vazyme, Nanjing, China) according to the manufacturer’s instructions. The purified products were submitted to Tsingke Biotechnology Co., Ltd. for Sanger sequencing.

### Genetic evolutionary analysis

Complete *P1* gene sequences were assembled from the sequencing data using DNASTAR software. The sequences were subsequently aligned with reference sequences retrieved from the National Center for Biotechnology Information database. MEGA 7.0 software was employed to construct phylogenetic trees using the neighbor-joining method with 1,000 bootstrap replicates, while comparing the verified DHAV-3 sequence with GenBank reference strains (Supplementary Table 1). Next, nucleotide and amino acid sequences of *P1, VP0, VP3*, and *VP1* were analyzed for identities with reference strains using DNASTAR. Subsequently, BioEdit software was used to analyze the amino acid mutations within the VP1 protein of GI clade strains. Finally, the three-dimensional structure of the isolate VP1 proteins was predicted using the SWISS-MODEL online tool.

## Results

### RT-PCR identification

Total RNA was extracted from liver samples of diseased ducks and reverse-transcribed into cDNA. The cDNA was subsequently amplified using DHAV-3-F and DHAV-3-R primers. Of the 103 tested samples, 14 yielded specific PCR amplicons of 720 bp, and Sanger sequencing confirmed that all were DHAV-3. The 14 isolates were named DHAV-3/JS/1-2401/2024 to DHAV-3/JS/14-2414/2024 (GenBank accession numbers: PX471080–PX471093). Detailed information is presented in Table 2.

**Table 2:** 14 DHAV-3 isolates information.

Isolate name	Localization	Age (d)
DHAV-3/JS/1-2401/2024	Lianyungang, Jiangsu, China	4
DHAV-3/JS/2-2402/2024	Lianyungang, Jiangsu, China	8
DHAV-3/JS/3-2403/2024	Lianyungang, Jiangsu, China	6
DHAV-3/JS/4-2404/2024	Huai’an, Jiangsu, China	21
DHAV-3/JS/5-2405/2024	Lianyungang, Jiangsu, China	18
DHAV-3/JS/6-2406/2024	Huai’an, Jiangsu, China	14
DHAV-3/JS/7-2407/2024	Lianyungang, Jiangsu, China	30
DHAV-3/JS/8-2408/2024	Lianyungang, Jiangsu, China	15
DHAV-3/JS/9-2409/2024	Suqian, Jiangsu, China	13
DHAV-3/JS/10-2410/2024	Yancheng, Jiangsu, China	2
DHAV-3/JS/11-2411/2024	Lianyungang, Jiangsu, China	26
DHAV-3/JS/12-2412/2024	Lianyungang, Jiangsu, China	15
DHAV-3/JS/13-2413/2024	Suqian, Jiangsu, China	10
DHAV-3/JS/14-2414/2024	Suqian, Jiangsu, China	10

### *P1* gene homology and phylogenetic analysis

The complete *P1* gene was successfully amplified from all 14 isolates using the primer pairs A-DHAV-3-F/A-DHAV-3-R and B-DHAV-3-F/B-DHAV-3-R, which yielded amplicons of 1,224 bp and 1,516 bp, respectively. Sequencing confirmed those assemblies as the complete *P1* gene sequences of DHAV-3.

All 14 isolates possessed a *P1* gene of 2,199 bp, which encoded a polyprotein of 733 amino acids. Nucleotide identity among isolates ranged from 95.8% to 100.0%. Compared with reference strains, isolates showed nucleotide identity ranging from 91.2% to 99.1%. The lowest identity (i.e., 91.2% to 91.5%) was with the Vietnamese strain DN2, thereby indicating a distant genetic relationship. At the amino acid level, identity among the isolates ranged from 99.3% to 100.0%, and identity with the reference strains ranged from 95.5% to 100.0%. The vaccine strain B63 showed the lowest amino acid identity (i.e., 95.5% to 95.9%) among all reference strains.

**Fig. 1:**
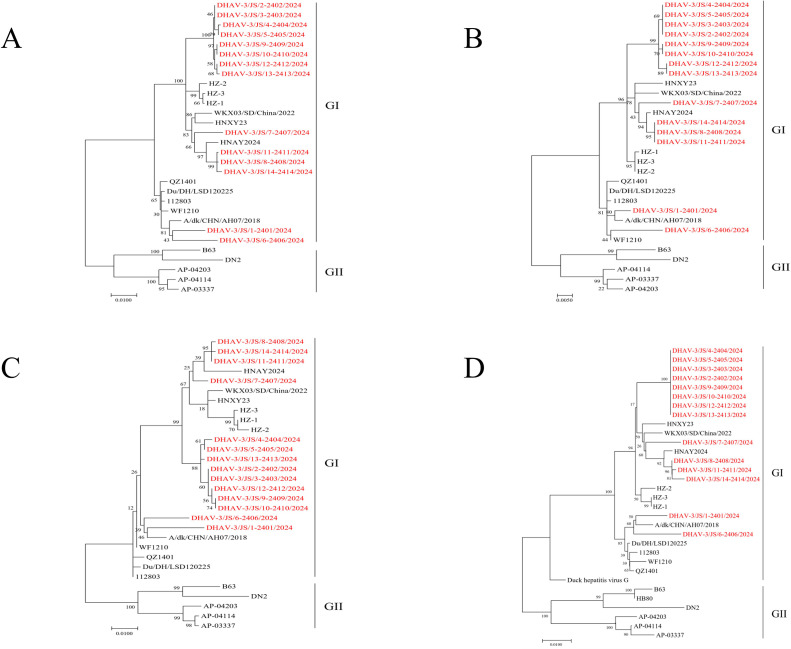
Phylogenetic trees of DHAV-3: (A) *P1* gene, (B) *VP0* gene, (C) *VP3* gene, and (D) *VP1* gene. All trees were constructed using the neighbor-joining method in MEGA 7.0 with 1,000 bootstrap replicates. Scale bars indicate genetic distances of 0.01 or 0.005 nucleotide substitutions per site. Isolates obtained in our study are highlighted in red.

A phylogenetic tree was constructed based on the *P1* gene sequences from the 14 isolates and 16 DHAV-3 *P1* gene sequences registered in GenBank (Fig. 1A). The 30 strains were divided into two clades: GI and GII. All isolates in our study, along with the majority of prevalent Chinese strains, were classified into GI. Among them, the isolates DHAV-3/JS/1-2401/2024 and DHAV-3/JS/6-2406/2024 were most closely related to the Anhui strain A/dk/CHN/AH07/2018 and clustered within the same subclade. Isolates DHAV-3/JS/7-2407/2024, DHAV-3/JS/8-2408/2024, DHAV-3/JS/11-2411/2024, and DHAV-3/JS/14-2414/2024 formed another subclade with Henan strain HNAY2024, whereas the remaining isolates constituted a separate subclade, thereby indicating close phylogenetic relationships among those isolates.

### *VP0* gene homology and phylogenetic analysis

Analysis of *VP0* sequences amplified from 14 isolates using DHAV-3-specific primers revealed that all sequences were 768 bp and encoded 256 amino acids. Nucleotide identity among the isolates ranged from 95.6% to 100.0%, and identity with the reference strains ranged from 92.3% to 99.6%. The lowest identity (i.e., 92.3% to 93.2%) was observed with the Vietnamese strain DN2. The amino acid identity among the isolates was 100.0%, which indicates high sequence conservation. The amino acid identity with reference strains ranged from 97.3% to 100.0%, with the lowest (i.e., 97.3%) observed with the vaccine strain B63.

The phylogenetic tree based on *VP0* gene sequences (Fig. 1B) showed that all strains were divided into two clades: GI and GII. GI contained all 14 isolates and all prevalent Chinese strains except for the vaccine strain B63. In particular, isolates DHAV-3/JS/1-2401/2024 and DHAV-3/JS/6-2406/2024 clustered into a subclade with Shandong strains (i.e., WF1210, QZ1401, and 112803) and Anhui strains (i.e., Du/DH/LSD120225 and A/dk/CHN/AH07/2018). Concurrently, isolates DHAV-3/JS/8-2408/2024, DHAV-3/JS/11-2411/2024, and DHAV-3/JS/14-2414/2024 clustered into the same subclade with the Henan strain HNAY2024, and DHAV-3/JS/7-2407/2024 also clustered into the same subclade as the above four strains. The remaining isolates clustered in the same subclade with close phylogenetic relatedness. GII included Korean strains, the Vietnamese strain DN2, and the vaccine strain B63, which showed distant phylogenetic relationships with the isolates in our study.

### *VP3* gene homology and phylogenetic analysis

Analysis of the *VP3* gene sequences from 14 isolates revealed that all isolates had a *VP3* gene length of 711 bp, encoding 237 amino acids. Nucleotide identity among the isolates was high (i.e., 95.1% to 100.0%), and identity with reference strains was lower (i.e., 90.6% to 98.6%). The lowest identity (i.e., 90.6% to 91.4%) was observed with the Vietnamese strain DN2. Amino acid sequences were highly conserved, with an amino acid identity among the isolates ranging from 99.6% to100.0%. In particular, isolate DHAV-3/JS/1-2401/2024 shared 99.6% amino acid identity with the other 13 isolates, and all remaining isolates shared 100.0% amino acid identity. The amino acid identity between the isolates and reference strains ranged from 97.0% to 100.0%, with the lowest identity (i.e., 97.0% to 97.5%) observed with the vaccine strain B63 and the Vietnamese strain DN2.

A phylogenetic tree constructed using *VP3* gene sequences from the 14 isolates and 16 DHAV-3 reference strains in GenBank (Fig. 1C) showed that all 30 strains were divided into two clades: GI and GII. All isolates belonged to GI. Among them, isolates DHAV-3/JS/1-2401/2024 and DHAV-3/JS/6-2406/2024 clustered with the Anhui strain A/dk/CHN/AH07/2018. Isolates DHAV-3/JS/7-2407/2024, DHAV-3/JS/8-2408/2024, DHAV-3/JS/11-2411/2024, and DHAV-3/JS/14-2414/2024 formed another subclade with the Henan strain HNAY2024. The remaining isolates clustered in the same subclade. By contrast, GII included Korean strains, the Vietnamese strain DN2, and the vaccine strain B63, all of which were phylogenetically distant from the isolates in our study.

### *VP1* gene homology, phylogenetic, and mutation analysis

The *VP1* gene sequences of all isolates were 720 bp and encoded a protein of 240 amino acids. Nucleotide identity among the isolates ranged from 95.6% to 100.0%, and identity with reference strains ranged from 89.9% to 99.4%. The lowest identity (i.e., 89.9% to 90.7%) was observed with the Vietnamese strain DN2, thereby indicating a distant genetic relationship. Amino acid identity ranged from 98.3% to 100.0% among the isolates and from 90.8% to 100.0% with reference strains, with the lowest identity (i.e., 90.8% to 92.1%) observed with the Korean strains AP-04114 and AP-03337.

The phylogenetic tree based on the *VP1* gene sequences (Fig. 1D) revealed division into two clades: GI and GII. GI included all isolates and all prevalent Chinese strains except the vaccine strain B63. Isolates DHAV-3/JS/7-2407/2024, DHAV-3/JS/8-2408/2024, DHAV-3/JS/11-2411/2024, and DHAV-3/JS/14-2414/2024 clustered in the same subclade as the Henan strains (i.e., HNAY2024 and HNXY23) and the Shandong strain WKX03/SD/China/2022. Isolates DHAV-3/JS/1-2401/2024 and DHAV-3/JS/6-2406/2024 formed a distinct subclade with the Anhui strain A/dk/CHN/AH07/2018, and the remaining isolates clustered in a separate subclade with relatively close phylogenetic relationships. GII comprised the Korean strains, the Vietnamese strain DN2, and the vaccine strain B63, all of which were phylogenetically distant from the isolates.

**Fig. 2:**
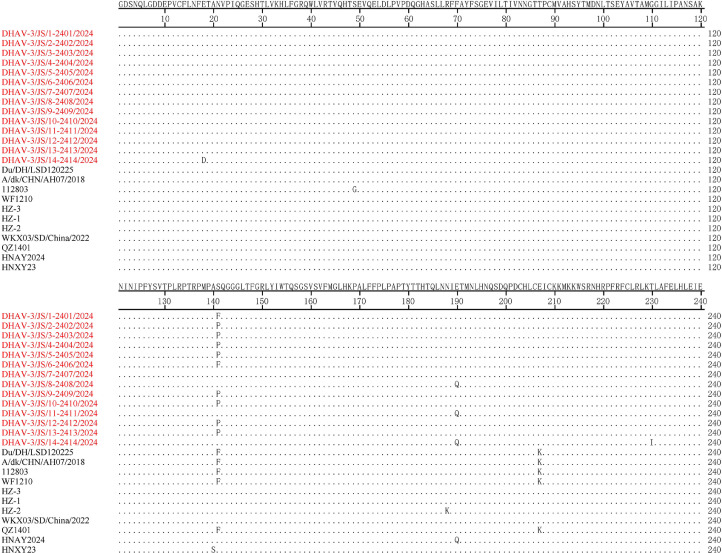
Multiple sequence alignment-based amino acid mutation analysis of the VP1 protein among DHAV-3 isolates in our study and GI reference strains. Isolates obtained in our study are highlighted in red.

VP1 protein analysis of 25 GI strains, including 14 isolates in our study and 11 reference strains from GenBank, revealed specific mutations (Fig. 2). Most amino acid sites were highly conserved, but some isolates had an S→P (or S→F) mutation at position 141 and an E→Q mutation at position 190, whereas some reference strains showed an S→F mutation at position 141 and an E→K mutation at position 207. Notably, the S→F mutation at position 141 was shared by isolates DHAV-3/JS/1-2401/2024, DHAV-3/JS/6-2406/2024, and reference strains Du/DH/LSD120225, A/dk/CHN/AH07/2018, WF1210, QZ1401, and 112803, which supports their close phylogenetic relationship. Similarly, the S→P mutation at position 141 was shared among isolates DHAV-3/JS/2-2402/2024, DHAV-3/JS/3-2403/2024, DHAV-3/JS/4-2404/2024, DHAV-3/JS/5-2405/2024, DHAV-3/JS/9-2409/2024, DHAV-3/JS/10-2410/2024, DHAV-3/JS/12-2412/2024, and DHAV-3/JS/13-2413/2024, thereby confirming their close genetic relatedness. Those mutational patterns corroborated the results of phylogenetic clustering.

**Fig. 3:**
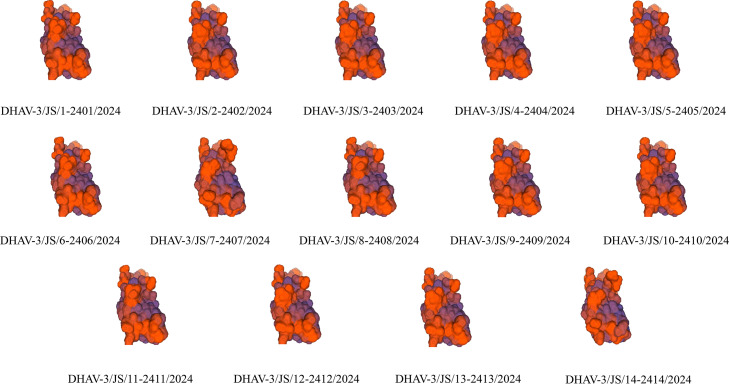
Three-dimensional structural prediction of the VP1 protein from 14 isolates obtained in our study. Structures are visualized with distinct coloration representing different structural domains.

In our study, VP1 protein structures of 14 DHAV-3 isolates were predicted (Fig. 3) and revealed highly conserved three-dimensional conformational features and functional domains across all isolates. That finding indicates that the core structure of VP1, a crucial structural protein, has remained stable during DHAV-3′s evolution, which is essential for preserving fundamental viral functions. However, structural variations observed in certain regions across different isolates are likely associated with genetic mutations occurring under diverse environmental conditions. Those variations may affect the antigenic epitopes of the VP1 protein and its binding capacity to host cells, which may influence viral pathogenicity, immunogenicity, and other biological properties.

## Discussion

In our study, 14 of 103 diseased duck samples from Jiangsu Province tested positive for DHAV-3, which confirmed its local prevalence. This finding is consistent with earlier reports from the region where DHAV-3 is endemic (Lin et al., 2016; Zhang et al., 2017; Wen et al., 2018). The observed detection rate was analyzed in detail, and the underlying factors are discussed below. First, samples were collected only from major duck-farming areas in Jiangsu, not covering the entire province, which may introduce geographical limitations and does not fully reflect the provincial-scale prevalence. Second, the etiology of liver disease in ducklings in Jiangsu is complex. In addition to DHAV-3, other pathogens such as DHAV-1 and duck astrovirus can induce similar pathological lesions. As the present study specifically targeted DHAV-3, the positive rate only reflects the detection rate of this virus in liver samples from diseased ducklings, rather than the overall prevalence of viral hepatitis in the region. Third, potential fluctuations in epidemic intensity during the sampling period may have influenced the overall detection rate. DHAV-1 is another major prevalent strain in mainland China. A systematic analysis of DVH in mainland China from 2009 to 2021 revealed a 12% incidence and 11% mortality, with DHAV-1 and DHAV-3 accounting for 38% and 49% of the tested samples, respectively (Zhou et al., 2022). These data, together with other studies, indicate ongoing shifts in the DHAV population structure, with DHAV-3 increasingly replacing DHAV-1 as the dominant strain in mainland China (Wen et al., 2018; Fehér et al., 2021; Ye et al., 2023; Zhao et al., 2024; Zhang et al., 2025).

Phylogenetic analysis of *P1, VP0, VP3*, and *VP1* consistently grouped all strains into two clades: GI and GII. GI contained all isolates and most Chinese strains, which is currently the predominant epidemic clade in Jiangsu Province and consistent with the global epidemic trend. The isolates shared high nucleotide homology with reference strains from Shandong, Anhui, Chongqing, and Henan Provinces. Whereas Jiangsu is geographically adjacent to Shandong, Anhui, and Henan, Chongqing is geographically distant. The difference implies that the inter-regional transmission of DHAV-3 is likely mediated not only through the intensive circulation of the duck industry among adjacent provinces but also via national-scale duck trade networks, breeding stock transportation, and feed commodity circulation that links geographically distant regions. GII comprised Korean strains, the Vietnamese strain DN2, and the Chinese vaccine strain B63. Both phylogenetic and identity analyses demonstrated a distinct evolutionary separation between the isolates and strains from South Korea and Vietnam, as evidenced by the notably low nucleotide identity (i.e., 89.9%) with the Vietnamese DN2 strain. Those findings, indicating clear geographical clustering in DHAV-3 genotypes, are consistent with published results from Xu et al. (2013) and Zhang et al. (2017). GII likely originated in South Korea before spreading to China and Vietnam (Ma et al., 2015). Such transnational transmission contributes to DHAV-3′s genetic diversity and increases the potential for recombination and accelerated evolution. Therefore, elucidating those cross-border transmission routes and enhancing international collaboration are crucial for effective control.

Comparative studies considering Picornaviridae indicate that variable regions in capsid proteins often localize to their termini, particularly the VP0 N-terminus and both termini of VP1 (Wang et al., 2008). That observation suggests that different DHAV capsid proteins perform specific conserved biological functions. VP0, a major immunogenic surface protein, is a known recombination hotspot between DHAV-1 and DHAV-3. Recombination in the region may alter viral surface conformation and thus potentially impair immune recognition and facilitate immune escape, which could compromise vaccine efficacy (Fehér et al., 2021). VP3, another major capsid surface protein harboring key antigenic epitopes, is a potential vaccine target (Tseng and Tsai, 2007; Zhou et al., 2022). VP1, the outermost and most variable protein in picornaviruses, is central to molecular epidemiology due to its high diversity (Kim et al., 2006). It contains B-cell and T-cell epitopes that induce neutralizing antibodies and plays a vital role in viral pathogenicity, evolution, and virulence determination (Jin et al., 2008;Gao et al., 2012; Xu et al., 2013). Therefore, analyzing *VP1* gene mutations provides critical insights into molecular evolution and the risks of antigenic drift or immune escape.

We found that the VP1 protein was highly conserved at most amino acid sites in both the isolates and reference strains. However, amino acid mutations were identified at positions 141 and 190 in some isolates, and mutations were observed at positions 141 and 207 in certain reference strains. Those mutations, resulting from genetic variation driven by factors such as selective pressure, could influence the protein’s structure and function. Notably, those mutations were not randomly distributed but exhibited specific patterns corresponding to phylogenetic clades. For instance, isolates and reference strains carrying the S141F mutation clustered into the same subclade, whereas isolates carrying the S141P mutation formed another distinct cluster. That finding suggests that these mutations may have been selectively retained along specific evolutionary paths. Cui et al. (2017) found that the antigenic epitopes of the DHAV-3 VP1 protein were primarily located between amino acid positions 90–175. The fragment showed more pronounced color development in response to duck anti-DHAV-3 serum than other regions, thereby indicating stronger immunogenicity, and was thus designated as the dominant antigenic region of the VP1 protein. The mutation at position 141 in our study occurred within the same region, which suggests that the amino acid mutation at the site may affect the pattern of antigen–antibody interaction and give the virus a stronger capability for immune escape. Position 141 was also defined as a hypervariable hotspot, indicating enhanced evolutionary plasticity (Zhao et al., 2025). Similarly, Xu et al. (2012) identified a highly variable region (i.e., amino acids 178–219) in DHAV-3 VP1 and suggested that high-frequency variants in the region may be associated with viral immune escape. The mutation at position 190 in our study localized to that critical region.

Vaccination is crucial for preventing DVH; therefore, increasing immunization coverage and conducting regular immunological monitoring of duck flocks are vital for control. Commercial DHAV-1 vaccines have resulted in an annual decline in the incidence of DHAV-1 and effectively controlled its spread. In 2023, China approved the first live attenuated DHAV-3 vaccine (i.e., strain HB80) (Fu et al., 2024), which offers a new strategy for control; however, DHAV-3 remains widespread due to its short market tenure. Notably, our study revealed a certain genetic distance between HB80 and the currently prevalent strains in Jiangsu. Phylogenetic analysis showed that all 14 isolates belong to the GI clade, whereas HB80 belongs to the GII clade. Sequence identity comparisons further confirmed this divergence, with nucleotide and amino acid identities ranging from 91.2% to 91.9% and 92.9% to 94.2%, respectively. Importantly, the challenge strain G (GenBank: EU755009) used in the development of HB80 was confirmed to belong to the GI clade, the same clade as the Jiangsu isolates. Fu et al. (2024) demonstrated that a single dose of HB80 conferred 96.7% protection against challenge with the G strain, indicating that despite the moderate genetic distance, HB80 can still provide effective cross-protection against GI clade strains. Accordingly, several strategies are recommended for regional prevention and control. These include the continued use of HB80 for basic immunization in Jiangsu and eastern China, given its demonstrated cross-protection against GI clade strains. Additionally, given the amino acid mutations identified at positions 141 and 190 in the VP1 protein of some Jiangsu isolates, monitoring these key sites in local isolates is recommended to assess their potential impact on neutralizing antibody binding. Furthermore, the development of a bivalent or chimeric vaccine based on local GI clade strains and HB80 may be considered to improve antigenic matching and enhance protective efficacy.

Currently, DHAV-3 is a significant pathogen threatening the duck industry, with an emerging variant challenging current diagnostic and control systems. Our findings elucidate DHAV-3′s molecular epidemiological characteristics. Phylogenetic analysis of *P1, VP0, VP3*, and *VP1* has enriched the DHAV-3 sequence database, clarified the geographic distribution of genetic lineages, and thus provided crucial data for monitoring viral evolution and refining vaccine strategies.

## Data availability

The *P1* gene sequences of the 14 DHAV-3 isolates obtained in our study have been deposited in the National Center for Biotechnology Information database (Accession ID: PX471080 to PX471093).

## Ethics statement

Our animal study was reviewed and approved by Shandong Agricultural University Animal Care and Use Committee (No. SDAUA–2024–231).

## Funding

This work was supported by the National Natural Science Foundation of China (grant number 32473019).

## CRediT authorship contribution statement

**Jiawen Yu:** Writing – original draft, Investigation. **Tingting Zhang:** Writing – original draft, Investigation. **Zitong Guo:** Investigation. **Yunwang Liu:** Software, Investigation. **Mengxue Yuan:** Software, Investigation. **Zijun Yang:** Resources, Investigation. **Xibo Wang:** Methodology, Investigation. **Xingdong Song:** Validation, Data curation. **Shijin Jiang:** Writing – review & editing, Project administration. **Liangmeng Wei:** Writing – review & editing, Project administration, Funding acquisition.

## Disclosures

The authors declare no conflicts of interest.

## Appendix Supplementary materials

Image, application 1
