# Supplementary material for: Identification and P1 genetic evolutionary analysis of duck hepatitis a virus type 3 in selected areas of Jiangsu, China in 2024
Source: Poult Sci. 2026 Mar 19;105(6):106824. doi: 10.1016/j.psj.2026.106824 (PMC13052077; doi:10.1016/j.psj.2026.106824)
Supplement: Supplementary file 1 [file mmc1.docx]

**Table S1.** Information regarding reference strains

| Strain | GenBank Accession No. | Time isolated | Host | Location |
| --- | --- | --- | --- | --- |
| Du/DH/LSD120225 | MK371024.1 | 2012 | Duck | Anhui, China |
| A/dk/CHN/AH07/2018 | MT767252.1 | 2018 | Duck | Anhui, China |
| 112803 | PQ777123.1 | 2011 | Anas platyrhynchos | Shandong, China |
| WF1210 | KP715492.1 | 2012 | Duck | Shandong, China |
| WKX03/SD/China/2022 | PP072258.1 | 2022 | Duck | Shandong, China |
| QZ1401 | KP715488.1 | 2014 | Duck | Shandong, China |
| HZ-3 | OP575306.1 | 2022 | Duck | Chongqing, China |
| HZ-1 | OP575304.1 | 2022 | Duck | Chongqing, China |
| HZ-2 | OP575305.1 | 2022 | Duck | Chongqing, China |
| HNAY2024 | PP977088.1 | 2024 | Duck | Henan, China |
| HNXY23 | OR666647.1 | 2023 | Duck | Henan, China |
| B63 | EU747874.1 | 2011 | Duck | Beijing, China |
| DN2 | JF914944.1 | 2009 | Duck | Viet Nam |
| AP-04114 | DQ812093.1 | 2003 | Duck | South Korea |
| AP-03337 | DQ256132.1 | 2003-2004 | Duck | South Korea |
| AP-04203 | DQ256134.1 | 2003-2004 | Duck | South Korea |
